# Small Object Tracking in LiDAR Point Clouds: Learning the Target-Awareness Prototype and Fine-Grained Search Region

**DOI:** 10.3390/s25123633

**Published:** 2025-06-10

**Authors:** Shengjing Tian, Yinan Han, Xiantong Zhao, Xiuping Liu

**Affiliations:** 1 School of Economics and Management, China University of Mining and Technology, Xuzhou 221116, China; tbh322@cumt.edu.cn; 2 DUT-BSU Joint Institute, Dalian University of Technology, Dalian 116024, China; xpliu@dlut.edu.cn; 3 School of Mathematical Sciences, Dalian University of Technology, Dalian 116024, China; 32001038@mail.dlut.edu.cn

**Keywords:** point clouds, LiDAR, small objects, visual tracking, deep neural network

## Abstract

Light Detection and Ranging (LiDAR) point clouds are an essential perception modality for artificial intelligence systems like autonomous driving and robotics, where the ubiquity of small objects in real-world scenarios substantially challenges the visual tracking of small targets amidst the vastness of point cloud data. Current methods predominantly focus on developing universal frameworks for general object categories, often sidelining the persistent difficulties associated with small objects. These challenges stem from a scarcity of foreground points and a low tolerance for disturbances. To this end, we propose a deep neural network framework that trains a Siamese network for feature extraction and innovatively incorporates two pivotal modules: the target-awareness prototype mining (TAPM) module and the regional grid subdivision (RGS) module. The TAPM module utilizes the reconstruction mechanism of the masked auto-encoder to distill prototypes within the feature space, thereby enhancing the salience of foreground points and aiding in the precise localization of small objects. To heighten the tolerance of disturbances in feature maps, the RGS module is devised to retrieve detailed features of the search area, capitalizing on Vision Transformer and pixel shuffle technologies. Furthermore, beyond standard experimental configurations, we have meticulously crafted scaling experiments to assess the robustness of various trackers when dealing with small objects. Comprehensive evaluations show our method achieves a mean Success of 64.9% and 60.4% under original and scaled settings, outperforming benchmarks by +3.6% and +5.4%, respectively.

## 1. Introduction

Single object tracking [1,2], a long-standing challenge in computer vision, provides essential environmental awareness for intelligent agents, facilitating advanced decision-making and interaction. In the past, 2D visual tracking with cameras has received extensive attention and achieved outstanding results based on deep neural networks [3,4,5,6]. Furthermore, with the development and popularization of Light Detection and Ranging (LiDAR) technology, the visual object tracking community has also sparked a wave of 3D single object tracking with point cloud, which has been attracting more and more attention in multiple domains, such as autonomous vehicles and mobile robotics.

Existing 3D single object tracking networks can be categorized based on their proposal generation techniques into point-based and bird’s eye view (BEV)-based approaches. Point-based methods extract seed points directly from the search area to estimate bounding boxes [7], whereas BEV-based methods transform points onto a bird’s eye view plane and subsequently leverage a 2D detection framework to forecast 3D bounding boxes. Prominent examples of BEV-based methods include V2B [8] and STNet [9]. Despite the inherent information loss due to projection in BEV-based methods, their adaptability in transitioning from 2D techniques and their potential for multimodal data integration render them highly valuable for exploration. However, within this domain, current methodologies predominantly concentrate on tracking standard-sized objects, neglecting the complexities associated with small objects, which remain an unexplored area.

To address this critical gap, this study shifts our efforts towards the prevalent and inevitable presence of small objects in real-world settings. It is widely acknowledged that pedestrians are often classified as small objects within standard datasets such as KITTI [10] and nuScenes [11]. In practice, we need to locate the specified object in 11.2 m × 7.2 m search regions, where a pedestrian, typically measuring just 1 m × 0.5 m, stands in stark contrast to a vehicle, which can extend over a larger area of more than 4 m × 2 m. Employing a voxel size of 0.3 m to discretize the search space, this relatively diminutive pedestrian would be converted into an object that corresponds to a mere 3 × 2 pixels in a 24 × 38 pseudo-image. This phenomenon strictly aligns with the established criteria for small objects as documented in the scholarly literature [12].

**Figure 1:**
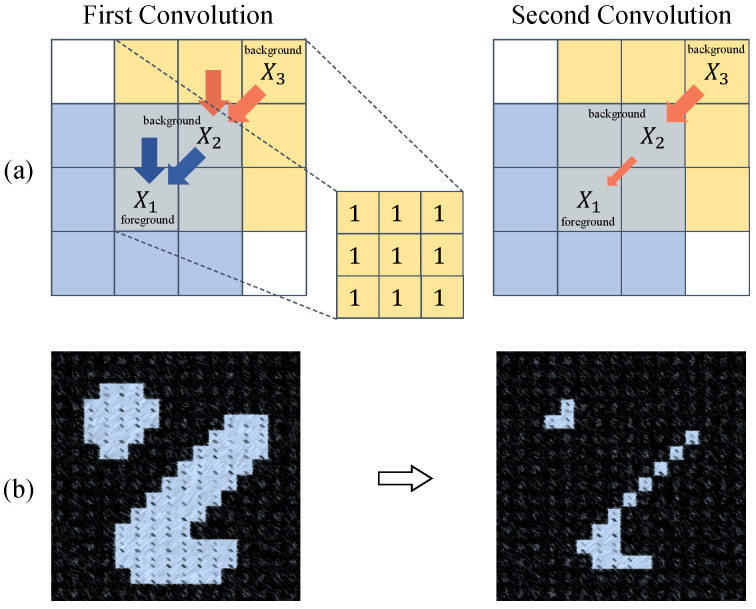
Erosion caused by convolution. (**a**) When performing the first convolution, the information in the blue area will converge to pixel $X_{1}$ along the direction of the blue arrow, and the information in the yellow area will similarly converge to $X_{2}$. When performing the second convolution, due to the aggregation of $X_{3}$ information by $X_{2}$, $X_{1}$ will also indirectly receive $X_{3}$ information. (**b**) The above convolutional layers diffuse features across pixels, causing the erosion of small object details. For a target, critical features (blue) are overwhelmed by the background (black) after convolution.

Addressing the challenge of 3D small object tracking, we encounter two primary obstacles for current deep learning-based methods: First, the sparsity and scarcity of foreground points across the entire scene result in features lacking the necessary discriminatory power. Second, small objects typically have narrow or pocket-sized bounding boxes, making them disproportionately sensitive to disturbances. The first barrier derives from two contributing factors. On one hand, LiDAR operates by emitting fan-shaped laser beams and collects intensity signals to form points upon contact with objects. Given the diminutive scale of small objects, these rays are less likely to intersect with the target, resulting in sparser foreground points. On the other hand, farthest point sampling is routinely employed to curtail the number of points during the refinement of high-level features. This method uniformly samples points across the entire space. However, since foreground points associated with small objects are confined to a limited region, this leads to an even more reduced count of foreground points post-sampling. As for the second obstacle, it engenders a heightened box sensitivity. Even minor deviations in the predicted box can precipitously lower the Success metrics, which are fundamentally tied to the Intersection-Over-Union (IoU) between the ground truth and predicted bounding boxes, and are susceptible to prediction biases. This necessitates a higher precision in the predicted boxes to maintain an acceptable Success metric. We have identified that a significant cause of inaccurate predictions is the information erosion effectuated by convolutional operations, as illustrated in Figure 1. Convolution enables the diffusion of information among pixels, which can erode the pivotal features of small objects. With small objects occupying a limited pixel area, their distinctive features are more readily overwhelmed by irrelevant features. Based on this analysis, the avenues for enhancing small object tracking can be encapsulated in two key strategies: increasing the density of foreground points and mitigating erosion induced by convolution.

**Figure 2:**
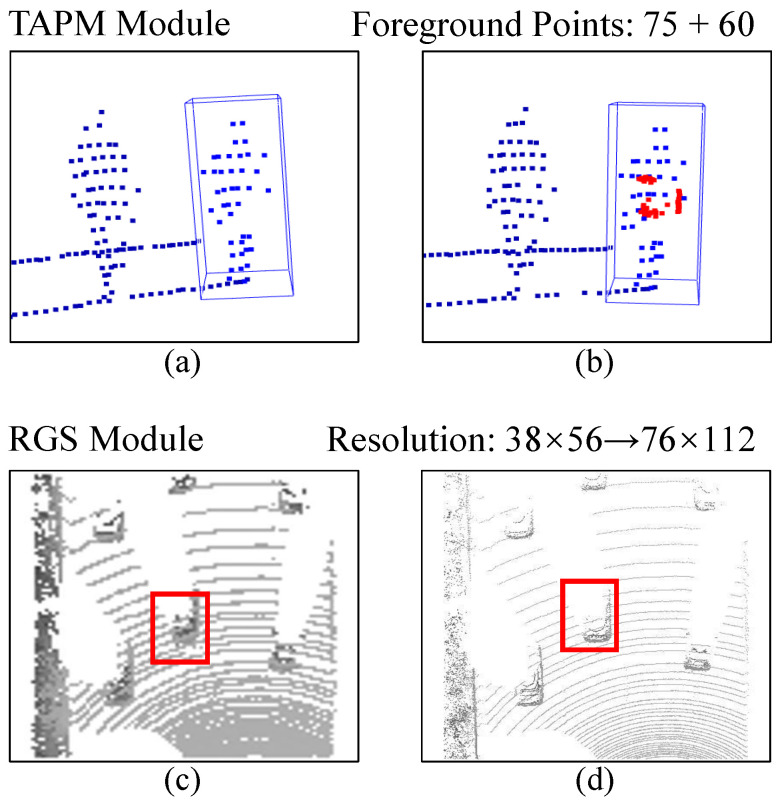
Illustrative diagram of the proposed module. (**a**,**b**) represent the point cloud of the target before and after using the target-awareness prototype mining module (TAPM). The blue boxes highlight objects of interest. The blue points in (**a**,**b**) are the original point cloud, and the red points in (**b**) are the recovered 60 foreground points. (**c**,**d**) shows the bird’s eye view map before and after using the regional grid subdivision module (RGS). The red boxes highlight objects of interest. Its resolution is increased from 38×56 to 76×112 one.

For the first principle, we delve into feasible optimization solutions. Recently, ISBNet [13] introduced an instance-wise encoder that replaces the farthest point sample with an instance-aware sample, ensuring the retention of foreground points. Although this sampling strategy could guarantee the density of foreground points, it cannot avoid information loss caused by down-sampling. Taking inspiration from the opposite perspective, we adopt the reverse operation of sampling to increase the density. To this end, we introduce a target-aware prototype mining module (see Figure 2) that leverages the reconstruction capabilities of a masked decoder to extract target prototype information across the entire feature space. This pioneering module fulfills a twofold role: it amplifies the count of foreground points and partially reconstructs the unique geometric details of the target. These enhancements bolster the distinctiveness of the foreground points, thereby aiding in the precise localization of small objects. As for the second enhancement, we incorporate a regional grid subdivision module (see Figure 2) designed to retrieve fine-grained features within the search area, capitalizing on Vision Transformer (ViT) technology and pixel shuffle layers. The experiments in Section 4.3 demonstrate that shrinking the voxel size during voxelization can enhance the tracking performance for small objects. Intuitively, this increases the resolution of the bird’s eye view. Higher-resolution images with clearer and richer details are conducive to tracking since more pixels can mitigate the information erosion effect. However, the reduction in voxel size will significantly increase computing resources. To balance effectiveness and efficiency, one may consider using sparse convolution [14,15]. It only performs convolution operations on non-empty pixels and avoids interference with invaluable pixels, but there are invalid pixels rather than non-empty pixels in bird’s eye view maps, which will absorb features of interfering substances or unrelated targets. Therefore, we design a regional grid subdivision module that lifts the low-resolution bird’s eye view map to a high-resolution one by a pixel shuffle layer [16]. Because the entire process resource consumption is concentrated in the voxelization step, our method can improve accuracy with minimal resource consumption. Finally, we summarize the function of the proposed modules in Figure 2, which simply demonstrates the advantages of our method.

Considering the experiment on the pedestrian category alone is not adequate to verify the robustness of the model against small targets, we elaborately design scaling experiments that scale other categories of objects into similar sizes with pedestrians to explore the adaptability of existing models to small objects. This work is devoted to giving an optimization method to improve the tracking performance of small objects. To our knowledge, this paper is the first to bring the concept of small objects into point cloud single object tracking.

In summary, our main contributions are threefold:

- We delineate the concept of small object tracking within LiDAR point cloud environments and scrutinize the unique challenges that small objects introduce to 3D single object tracking. To effectively track small objects, our model addresses the sparse distribution of foreground points and the feature degradation resulting from convolutional operations.
- We introduce two innovative modules: the target-awareness prototype mining (TAPM) module and the regional grid subdivision (RGS) module. The TAPM module adeptly enhances the density of foreground points without compromising information integrity, while the RGS module mitigates feature erosion without imposing additional computational demands.
- We devise a scaling experiment to evaluate and compare the robustness of diverse tracking methods when confronted with small objects. Our approach has yielded remarkable outcomes in standard as well as scaled experimental conditions.

The remainder of this paper is organized as follows: Section 2 reviews related work and identifies gaps in small object tracking. Section 3 details our methodology, including the TAPM and RGS modules. Section 4 presents experiments and comparisons. Section 5 discusses limitations, and Section 6 concludes with future directions.

## 2. Related Work

### 2.1. 3D Single Object Tracking

SC3D [1] marks the pioneering effort in employing deep neural networks for point cloud single object tracking. It assesses the similarity score between candidates and a template, opting for the candidate with the maximum score as the final output. However, its results are subject to the generation of candidates that cannot be integrated into the whole pipeline in an end-to-end manner. Subsequently, P2B [7] formulates an end-to-end framework that embeds template features into search features and feeds the fused features into a VoteNet [17] to generate predicted bounding boxes. P2B has established itself as a seminal work, inspiring subsequent research paradigms. MLVSNet [18] adopts Hough voting on multi-level features to adjust targets at different scales. Treating the ground truth bounding box given in the first frame as a strong cue, BAT [19] improves the information embedding processing by geometric prior, assisting the model in learning more accurate features. Observing the sparse issue in the voting-based head for predicting the bounding box, V2B [8] replaces the voting-based detection head with a BEV-based one, which projects search points onto a dense bird’s eye view feature map to tackle the sparsity of point clouds. With the popularity of transformers [20] in computer vision, LTTR [21], PTT [22], PTTR [23], Trans3DT [24], and nSTNet [9] attempt to embed the attention module into the original model.

Recently, to fully take advantage of the background, some methods have arisen that consume the whole template instead of a target-only template. M2Track [25] and CXTrack [26] start using the complete previous frame to enhance the feature fusion process. M2Track initially generates coarse bounding boxes by estimating the relative motion between consecutive frames, followed by a refinement process in the subsequent stage to achieve a high-precision bounding box. In contrast, CXTrack employs a sophisticated information embedding module that directly processes the point cloud data from both the preceding and current frames. The system commences by accurately predicting the centroids of the bounding boxes and segmenting the foreground. Building upon this, it utilizes the transformer-based X-RPN module to enhance the predictions by harnessing the consolidated features from the prior module. MBPTrack [27] has taken advantage of the memory mechanism to improve 3D Point Cloud Tracking. Wu et al. [28] proposed exerting the powerful representation of the pre-trained 2D foundation trackers and thus designed a 3D-to-2D projection module.

Current state-of-the-art methods predominantly employ transformer-based architectures, which concurrently integrate feature extraction and semantic embedding mechanisms. However, these approaches exhibit suboptimal performance in small-target tracking scenarios due to inherent limitations, including sparse foreground feature distribution and progressive information erosion in convolutional operations. While hierarchical encoder architectures combining global contextual modeling and local structural representation demonstrate notable empirical performance for generic object tracking, they exhibit persistent limitations in small object tracking efficacy, particularly under conditions of low feature density and spatial degradation. To mitigate these challenges, we propose a dual-module framework comprising a Target-Aware Prototype Matching (TAPM) mechanism and a Recurrent Gradient Supervision (RGS) module, designed to enhance discriminative feature preservation and mitigate gradient-based information loss in small-target tracking paradigms.

### 2.2. Small Objects Researches

In the field of 2D visual conception, small objects have always been a challenging problem in detection and tracking [29,30]. We summarize some prevalent and key methods for small objects from three perspectives: multi-scale feature learning, context-based methods, and data augmentation.

Multi-scale Feature Learning. Such methods recon that small object features are prone to be masked by other object features. To highlight the small object features, researchers proposed the feature pyramids network [31], which aggregates information in different feature layers thereby multi-scale features could produce an effect equally. Although PANet [32], AugFPN [33], TridentNet [34], and many other similar methods are effective, the difference lies in the details of the model rather than the central idea.

Context-based Methods. Due to the inherent characteristics of small objects, it is challenging to obtain sufficient information from the target. Therefore, some methods look forward to assisting tasks with inter-object relationships. ION [35] integrates contextual information outside the region of interest (ROI) using spatial recurrent neural networks. PyramidBox [36] proposes a novel context-assisted single-shot face detector. Relation Networks [37] designs an object relation module that processes a set of objects simultaneously through the interaction between their appearance feature and geometry.

Data Augmentation. This kind of method can be divided into two categories. One is traditional data augmentation, which increases the frequency of tiny targets in the training phase to deal with small objects. In Stitcher [38], images are resized into smaller components and then stitched into the same size as regular images. Augmentation [39] proposes to oversample those images with small objects and augment each of those images by copy–pasting small objects many times. The other is the GAN-based method, which utilizes GAN to generate high-resolution images or high-resolution features. In SOD-MTGAN [40], the generator is a super-resolution network that can up-sample small blurred images into fine-scale ones and recover detailed information for more accurate detection. Perceptual GAN [41] learns more discriminative features by lifting small objects to super-resolved ones, avoiding the computational cost of multi-scale feature learning. BFFBB [42] proposes a novel feature-level super-resolution approach. It not only correctly addresses these two desiderata but is also integrable with any proposal-based detectors with feature pooling.

There are also some special designs according to the characteristics of the vision tasks. S3FD [43] aims to solve the problem of anchor-based face detection, which deteriorates dramatically as objects get smaller, by paying special attention to small faces at different stages of training. Feedback-driven [44] uses the loss distribution information as the feedback signal guiding the gradient feedback process. For the object tracking task, Liu et al. [45] proposed a saliency-based aggregation signature that can be viewed as a feature descriptor to deal with vague and variable appearances and lens blur. SmallTrack [46] introduces a wavelet pooling layer and graph-enhanced module for aerial object tracking. Note that the above methods are mainly for small objects in 2D vision, whereas this study deals with 3D LiDAR point clouds, which are not suitable for direct lifting by these 2D methods.

## 3. Methodology

### 3.1. Problem Definition

Given a point cloud of the first frame with the bounding box of an arbitrarily specified object (template), 3D single object tracking aims to locate the designated target in the following frames. Most prevailing tracking methods comply with the Siamese paradigm by which the target will be retrieved with the template cropped from the first frame. The task can be cast as finding a Tracker meeting with Equation (1),

(1) $$Tracker\left(P_{S},P_{T}\right)=B_{S},$$

where $P_{T}$ denotes the set of template points. $P_{S}$ and $B_{S}$ denote the set of search region points and the bounding box of the target in the current frame, respectively. In addition, the physical size of the target usually remains unchanged in the 3D space, thus the bounding box is represented by B=(x,y,z,θ), which consists of the coordinate of the box center (x,y,z) and the orientation angle θ around the up-axis. Specifically, the search region points $P_{S}\in R^{N_{S}\times 3}$ and the template points $P_{T}\in R^{N_{T}\times 3}$ will be fed into a shared encoder to acquire individual geometric features $F_{S}\in R^{N_{S}\times C}$ and $F_{T}\in R^{N_{T}\times C}$. Then, the template features will be embedded into search features by a relation modeling module to generate fusion features ${\tilde{F}}_{S}\in R^{N_{S}\times C}$. Finally, a detection head will utilize fusion features to predict the final results. Here we focus on the BEV-based detection head, which projects the features into bird’s eye view maps $V\in R^{H\times W\times C}$ by voxelization and pooling operation and then uses a series of 2D convolution layers to predict Hot Map $H\in R^{H\times W\times 1}$, Offset $O\in R^{H\times W\times 3}$, and Z-axis $Z\in R^{H\times W\times 1}$ separately as described in V2B [8].

As discussed in Section 1, existing methods ignore challenges incurred by small objects. To this end, we propose two effective modules: the target-awareness prototype mining module and the regional grid subdivision module. We will illustrate the implementation of two designed modules in Section 3.2 and Section 3.3, respectively. Moreover, details and the loss functions will be given in Section 3.4.

### 3.2. Target-Awareness Prototype Mining

**Figure 3:**
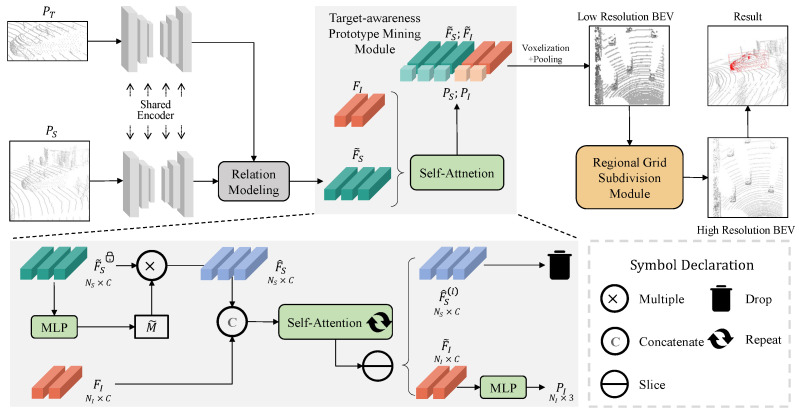
The overall architecture of the proposed method. The template point cloud and the search region point cloud are fed into a shared encoder to generate respective geometric features. Then the template features are embedded into search region features by the relation modeling module. Subsequently, the target-awareness prototype mining module will highlight the foreground features and enrich them through several self-attention layers. Finally, the target position is predicted by a BEV-based detection head accompanied by the regional grid subdivision module. On the top-right corner of this figure, the tracked object is represented by red points which are covered by red bounding box.

To highlight the presence of the target in the search region, we propose the target-awareness prototype mining (TAPM) module, which enhances foreground saliency through two mechanisms: masked features reconstruction and prototype features mining. The former learns to prioritize target geometry by reconstructing masked regions. The latter aims to amplify target-aware features and suppress background, promoting to generate completion points. Specifically, as Figure 3 shows, template points $P_{T}$ and search region points $P_{S}$ are firstly fed into a shared encoder to generate their respective features, and then they will pass through the relation modeling module to obtain fusion features ${\tilde{F}}_{S}$.

For masked features reconstruction, the fusion features ${\tilde{F}}_{S}$ will be sent into the TAPM module accompanied by learnable substrate features $F_{I}\in R^{N_{I}\times C}$. Then, a multi-layer perceptron (MLP) is employed to generate the mask $\tilde{M}\in R^{N_{S}\times 1}$ and $\tilde{M}$ multiply as weight into fused features to obtain enhanced fusion features ${\hat{F}}_{S}\in R^{N_{S}\times C}$ as Equation (2) shows,

(2) $$\tilde{M}=\sigma \left(FW^{T}+b\right),{\hat{F}}_{S}=\tilde{M}\cdot {\tilde{F}}_{S},$$

where σ is the Sigmoid function and · is the element-wise product operation. The value of this step is to enhance the features of the foreground points and weaken the features of the background points. It is worth noting that we cut off the gradient feedback process from ${\tilde{F}}_{S}$ because we intend to regard it as a wizard to guide substrate features $F_{I}$ transformation to prototype features without affecting the original feature fusion process.

To learn prototype features of the target, the concatenation of substrate features $F_{I}$ and fusion features $\hat{F_{S}}$ will pass through self-attention layers and iterate *l* times:

(3) $$\left[{\hat{F}}_{S}^{\left(i\right)},F_{I}^{\left(i\right)}\right]=\mathrm{Attn}\left(\left[{\hat{F}}_{S}^{\left(i+1\right)},F_{I}^{\left(i+1\right)}\right]\right),$$

where 1≤i≤l, [,] means concatenate operation, ${\hat{F}}_{S}^{0}={\hat{F}}_{S},F_{I}^{0}=F_{I}$.

Leveraging a series of self-attention layers, substrate features repeatedly interact with fusion features and finally transfer into prototype features ${\tilde{F}}_{I}=F_{I}^{\left(l\right)}$. Finally, the teacher features ${\hat{F}}_{S}^{\left(l\right)}$ will be dropped after completing their role of guidance, and the prototype features ${\tilde{F}}_{I}$ will be used to predict the coordinates of completion points $P_{I}\in R^{N_{I}\times 3}$ by the MLP layers. The enhanced point cloud $\left[P_{S},P_{I};{\tilde{F}}_{S},{\tilde{F}}_{I}\right]$ will be sent to downstream tasks. To ensure that the prototype points fall on the target, we introduce Chamfer Distance (CD) loss constraint coordinates, as detailed in Section 3.4.

### 3.3. Regional Grid Subdivision

Small objects are sensitive to prediction bias, so they require more precise positioning than general objects to obtain outstanding results. One of the important reasons for positioning deviation is the information dilution brought by convolution. It has been proven in the field of traditional vision that the higher the resolution, the stronger the robustness of the image to erosion. Inspired by this, we insert a regional grid subdivision (RGS) module into the original BEV-based detection head.

**Figure 4:**
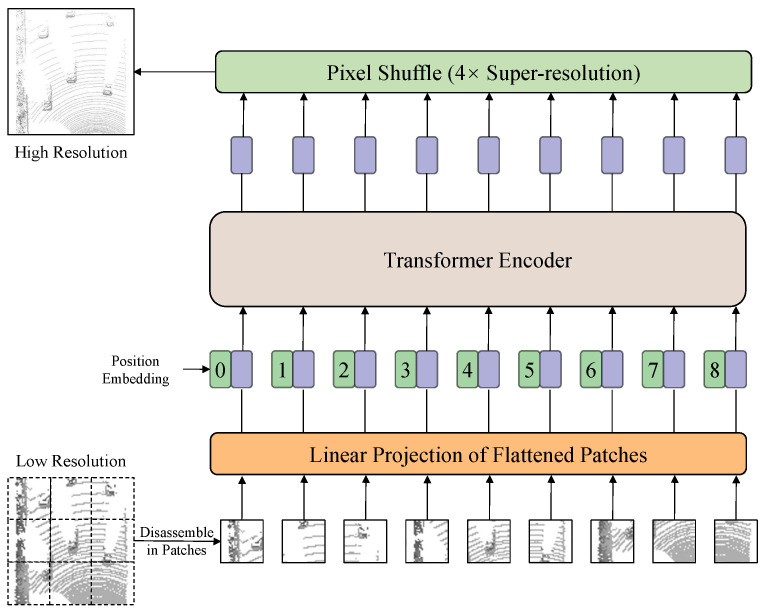
The architecture of the regional grid subdivision module, which consists of a ViT layer and a pixel shuffle layer. Firstly, the bird’s eye view feature map decouples into patches, and the patches will be flattened and added with position embedding. They will interact and generate reconstructed features by a series of transformer encoders. Finally, each 128-channel pixel feature is divided into four 32-channel pixel features by the pixel shuffle layer.

As shown in Figure 4, the RGS module is composed of two subparts: a ViT layer [47] and a pixel shuffle layer [16]. At first, the enhanced point cloud $\left[P_{S},P_{I};{\tilde{F}}_{S},{\tilde{F}}_{I}\right]$ is projected into a bird’s eye view feature map $V\in R^{H\times W\times C}$ by voxelization and pooling. Then this map will be patched based on pixels and sent to a ViT layer consisting of position embedding and a transformer encoder. Finally, a pixel shuffle layer is employed to generate a high-resolution BEV feature map $\tilde{V}\in R^{2H\times 2W\times \frac{C}{4}}$. After the RGS module, the resulting $\tilde{V}$ will be passed through a series of 2D convolutional layers to generate the final result, which consists of Hot Map $H\in R^{H\times W\times 1}$, Offset $O\in R^{H\times W\times 3}$, and Z-axis $Z\in R^{H\times W\times 1}$. The introduction of the ViT layer has two aspects of significance. On the one hand, it reorganizes pixel features into channels that make it easier to separate detailed features from the overall during conducting sub-pixel. On the other hand, the ViT layer helps pixel features capture global information and compensates for the lack of global information caused by increased resolution.

### 3.4. Loss Functions

Following STNet [9], we employ focal loss $L_{hm}$, smooth-L1 loss $L_{off}$, and $L_{z}$ to constrain H, O, Z in the first stage, respectively. At first, some variables need to be declared. The real coordinate of the target center is (x,y,z). θ indicates the ground truth rotation angle. *v* is the voxel size and $\left(x_{min},y_{min}\right)$ indicates the minimum coordinate of search regions. $c=\left(c_{x},c_{y}\right)$ is the 2D target center in the X–Y plane, where $c_{x}=\frac{x-x_{min}}{2v}$ and $c_{y}=\frac{y-y_{min}}{2v}$. The discrete 2D center $\tilde{c}=\left({\tilde{c}}_{x},{\tilde{c}}_{y}\right)$ is defined by ${\tilde{c}}_{x}=\left\lfloor c_{x}\right\rfloor$ and ${\tilde{c}}_{y}=\left\lfloor c_{y}\right\rfloor$, where $\left\lfloor .\right\rfloor$ means rounding down. Then focal loss $L_{hm}$ between H and the ground truth $H^{gt}$, the smooth-L1 loss $L_{off}$ between O and the ground truth $O^{gt}$, and the smooth-L1 loss $L_{off}$ between Z and the ground truth $Z^{gt}$ can be calculated, where $H_{ij}^{gt}=1$ if $\left(i,j\right)=\tilde{c}$ otherwise $\frac{1}{1+∥\left(i,j\right)-\tilde{c}∥}$, $O_{ij}^{gt}=\left[\left(i,j\right)-c,\theta \right]$, and $Z_{ij}^{gt}=z$. In addition, to ensure that the prototype points are target-aware, we use Chamfer Distance (CD) loss $L_{cd}$ to constrain interpolation points $P_{I}$ as shown in Equation (4).

(4) $$\begin{array}{c} L_{cd}=\sum_{p_{T}^{i}\in P_{T}}\min_{p_{I}^{j}\in P_{I}}{∥p_{T}^{i}-p_{I}^{j}∥}_{2}^{2}+\sum_{p_{I}^{i}\in P_{I}}\min_{p_{T}^{j}\in P_{T}}{∥p_{I}^{i}-p_{T}^{j}∥}_{2}^{2}. \end{array}$$

Herein, $P_{T}$ is a template point cloud aligned with the current frame target through translation and rotation. Finally, we aggregate all the mentioned losses as our final loss,

(5) $$\begin{array}{c} L=\lambda_{1}\left(L_{hm}+L_{off}\right)+\lambda_{2}L_{z}+\lambda_{3}L_{cd}, \end{array}$$

where $\lambda_{1}=1$, $\lambda_{2}=2$, $\lambda_{3}=1\times 10^{-6}$ for non-rigid objects and $\lambda_{3}=2\times 10^{-7}$ for rigid objects.

## 4. Experiments

### 4.1. Experimental Settings

Dataset. We conducted extensive experiments on the KITTI (http://www.cvlibs.net/datasets/kitti/eval_tracking.php, accessed on 2 June 2025) [10] and nuScenes (https://www.nuscenes.org/download, accessed on 2 June 2025) [11] datasets. The KITTI contains 21 training video sequences and 29 test sequences. We followed SC3D [1] and split the training sequences into three parts: 0–16 for training, 17–18 for validation, and 19–20 for testing. For the nuScenes, we used its validation split to evaluate our model, which contains 150 scenes. In addition, to compare the robustness of existing methods to small objects, we also proposed a new scaling experimental setup on KITTI. All experiments were conducted with TITAN GPUs.

Implementation Details. For the 3D single object tracking task, we enlarged the ground truth bounding box in the current frame by 2 meters to obtain the sub-region and sample 1024 points from it as $P_{S}$. Simultaneously, we cropped and aggregated the target point clouds of the first and previous frames, and then sampled 512 points from the aggregated points as templates $P_{T}$. For the shared encoder and relation modeling module, we adhered to the same configurations as employed in STNet [9]. Within the target-awareness prototype mining module, we set the prototype point number equal to 64 and executed self-attention iterations 5 times. For the detection head, we set the voxel size equal to 0.2m and performed 4× super-resolution on bird’s eye view feature maps.

Evaluation Metrics. We followed one pass evaluation (OPE) [48] to evaluate all the methods, which consists of two metrics: Success and Precision. The Success and Precision metrics are computed as follows. The Success metric measures the overlap accuracy between the predicted and ground truth bounding boxes across all frames. Four steps are contained: (1) For each frame, compute the Intersection-over-Union (IoU) between the predicted BBox and the ground truth BBox. (2) Vary the IoU threshold (denoted as τ) from 0 to 1 in increments (e.g., 0.01). For each τ, compute the proportion of frames where IoU ≥τ. (3) Plot these proportions (Y-axis) against τ (X-axis). (4) Compute the Area Under the Curve (AUC) of the Success Plot using numerical integration. A higher AUC indicates better tracking robustness.

Precision metric measures the accuracy of the predicted box center location relative to the ground truth. Four steps are contained: (1) For each frame, compute the Euclidean distance (ED) between the centers of the predicted and ground truth bounding boxes. (2) Vary the distance threshold (denoted as ϵ) from 0 to 2 meters in increments (e.g., 0.1 m). For each ϵ, compute the proportion of frames where ED ≤ϵ; (3) Plot these proportions (Y-axis) against ϵ (X-axis). (4) Compute the AUC of the Precision Plot. A higher AUC indicates better localization accuracy.

Scaling experiment. Given a scene point cloud, we extracted the foreground points based on the bounding box. We marked the set of foreground points as $P_{f}$ and the background as $P_{b}$. The set of foreground points can be represented by Equation (6)

(6) $$P_{f}={\left\{p_{i}=\left(x+\Delta x_{i},y+\Delta y_{i},z+\Delta z_{i}\right)\right\}}_{i=1}^{N_{f}}$$

where (x,y,z) is the coordinates of the center point of the bounding box. After scaling, the new foreground point set will be defined as Equation (7) and the set of background points $P_{b}$ remains,

(7) $${\tilde{P}}_{f}={\left\{p_{i}=\left(x+r\Delta x_{i},y+r\Delta y_{i},z+r\Delta z_{i}\right)\right\}}_{i=1}^{N_{f}}$$

where r∈(0,1) is the scaling rate parameter.

### 4.2. Results

Experiments under scaling settings. We retrained P2B [7], BAT [19], V2B [8], STNet [9], and M2Track [25] under scaling setting and the results are reported in Table 1. After scaling, a notable decline in the Success metric was observed across all methods, aligning with the heightened box sensitivity typically associated with small objects. By jointly analyzing this table, it is not difficult to find more performance degradation in point-based methods (P2B, BAT, M2Track). The reason is that such methods require selecting candidates through farthest point sampling; however, scaling shrinks the covering volume of the target and makes it more difficult to obtain points that fall on the targets. Compared to point-based methods, BEV-based methods perform more stably, as they do not require sampling during the prediction phase. Our method is 3.6 Success higher than the benchmark (STNet) under the original setting, and the gap widens to 5.4 when migrating to the scaling setting. This fact reflects that our method is more robust than the benchmark.

Experiments on the KITTI dataset. Except for scaling experiments, non-scaling experiments are also conducted to validate whether our method can maintain strong performance on normal categories. Therefore, we provide a comprehensive comparison of the proposed method against state-of-the-art 3D single object trackers, including SC3D [1], P2B [7], 3DSiamRPN [49], LTTR [21], MLVSNet [18], BAT [19], PTT [22], V2B [8], PTTR [23], STNet [9], M2-Track [25], and Trans3DT [24]. As illustrated in Table 2, the average Success of our methods exceeds all the above. Compared with the benchmark (STNet), our method has significantly improved performance in the pedestrian category, which can be attributed to the specialized optimization of small objects. Unlike M2Track, which belongs to motion-based methods, our method is still based on geometric shape matching. Therefore, while our method may exhibit slightly lower performance in the pedestrian category compared to M2Track, its performance on rigid objects with clear geometric structures is significantly better than M2Track.

Experiments on the nuScense dataset. To compare the generalization ability, we tested the KITTI pre-trained model on the nuScenes dataset. Unfortunately, due to the sensitivity of the target-awareness prototype mining module to changes in distribution, our method did not achieve state-of-the-art results as Table 3 shows. The reason may lie in two aspects. Our method excels in small object tracking while nuScenes focuses on the normal setting. Moreover, the TAPM module, trained on 64-beam LiDAR, struggles with nuScenes 32-beam data due to sparser point clouds. Future work will explore domain adaptation techniques to improve cross-dataset generalization.

**Figure 5:**
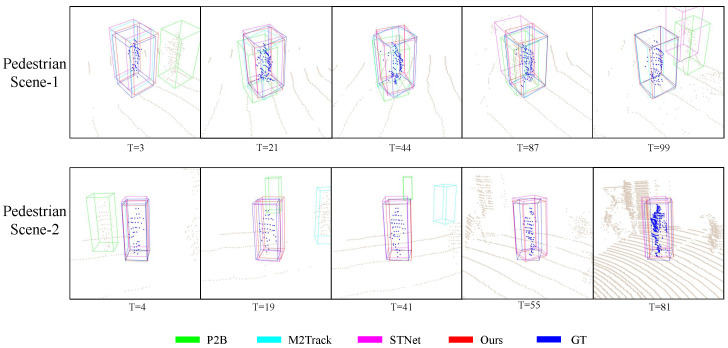
Visualizationresults of the pedestrian category. We selected two typical sequences in the KITTI datasets to compare the tracking results of different methods.

Visualization. We compare the performance between different methods qualitatively. Comparing the two sets of pictures in Figure 5, when there is no intra-class distractor, most methods can locate the target accurately. However, when there are intra-class distractors, P2B and STNet are misled by the bystander in “Ped Scene-1”, while P2B and M2Track are misled in “Ped Scene-2”. In addition, although both STNet and ours locate targets successfully in “Ped Scene-2”, our bounding boxes are more closely aligned with the ground truth.

**Figure 6:**
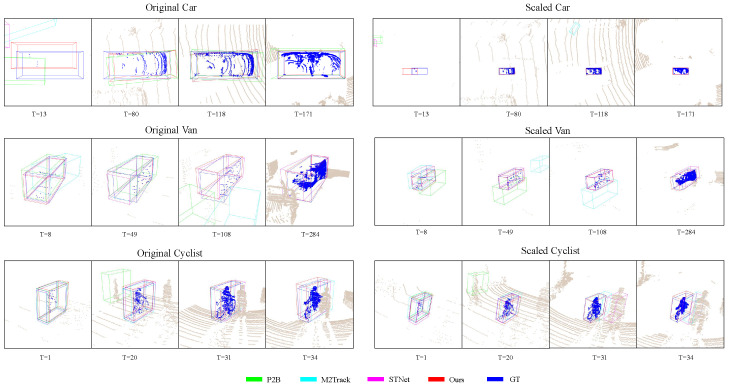
Visualizationresults of scaling experiments. The same scenes were selected before and after scaling, so by comparing the two groups of images on the left and right we can observe the difference in the effect before and after scaling.

Comparing the results between the two experimental settings in Figure 6, we can see that the proposed approach has two major advantages. One is that our method can effectively deal with sparsity. For the first scene (Car) in the first row, the foreground points are extremely sparse in the 13th frame (T = 13), but in such an extreme case, our method still successfully seeks the target out while other methods fail. The other, which is also the main contribution of this paper, is that our approach is more robust for small objects than other trackers. For “Car Scaled”, other methods lose the target in the first few frames, which makes it impossible to track the target even if the point cloud becomes dense in subsequent frames. For “Van Scaled”, STNet and our method maintain superiority while point-based methods are quite confused about where the target is. For “Cyclist”, all methods have achieved remarkable results except P2B under the original setting while STNet and M2Track obtain failure cases caused by the disturbance under the scaling setting. We assume that the robustness of our method to intra-class distractors comes from the prototype mining ability of the TAPM module to complete and recognize the target.

**Figure 7:**
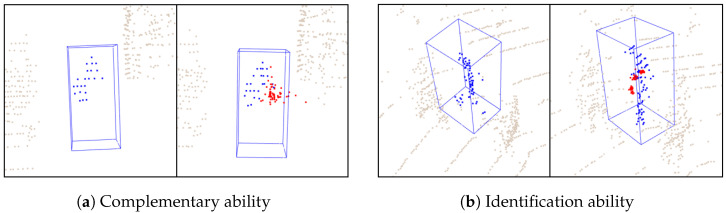
Visualization of interpolation points effects. The interpolation points generated by TAPM can restore the shape of the target. More importantly, it can correctly identify the target from intra-class distractors. The blue points in are the original point cloud, and the red points are the recovered foreground points.

To further validate the above findings, Figure 7 presents some visualization results. In particular, as shown in Figure 7a, the TAPM module can reconstruct the right part of the body of the pedestrian. From Figure 7b, it can accurately complete some points around the target without being misled by other pedestrians.

### 4.3. Ablation Studies

To explore the factors that affect the model results, we conducted ablation experiments on the KITTI dataset.

Voxel size. Voxel size is the side length of the cube space occupied by each voxel during voxelization. Increasing the value of voxel size will encode the space into a higher resolution volume, and it will increase the resolution of the bird’s eye view map which is obtained by pooling the volume. As discussed in Section 1, decreasing the voxel size can relieve convolutional erosion and improve accuracy. Table 4 shows the gap in average tracking accuracy between STNet and our method under different voxel sizes. When the voxel size of STNet decreased from 0.4 m to 0.2 m, the mean Success increased from 58.3 to 61.3, and among the most obvious benefits was the pedestrian category. However, as the resolution increased, the performance of STNet in the Van category degraded. This may be because smaller voxel units do not efficiently encode the detailed features of larger objects. In contrast, our method solves this problem by adding the vit layer to make the network more powerful in encoding pixel features. In addition to the comparison of effects, the video memory consumed by the training model cannot be ignored. Although STNet-0.2 can train with 84 batches (ours-0.2 is 42) at a time, the accuracy was much lower than ours, and the computation amount increased exponentially when the voxel size was reduced to 0.1. STNet-0.1 can only train with 27 batch sizes, which made it very difficult for STNet to achieve our accuracy by continuing to reduce the voxel size.

Model components. We investigate the effectiveness of the proposed module with Table 5 on the pedestrian category. It is noteworthy that adding only a pixel shuffle layer will lead to side effects. That is because pixel shuffle splits one 128-channel pixel feature into four 32-channel pixel features so that the structure of each pixel’s corresponding location cannot be efficiently encoded. After adding the ViT layer, which brings more powerful coding capability, the pixel shuffle layer will be a boost to the whole network.

**Figure 8:**
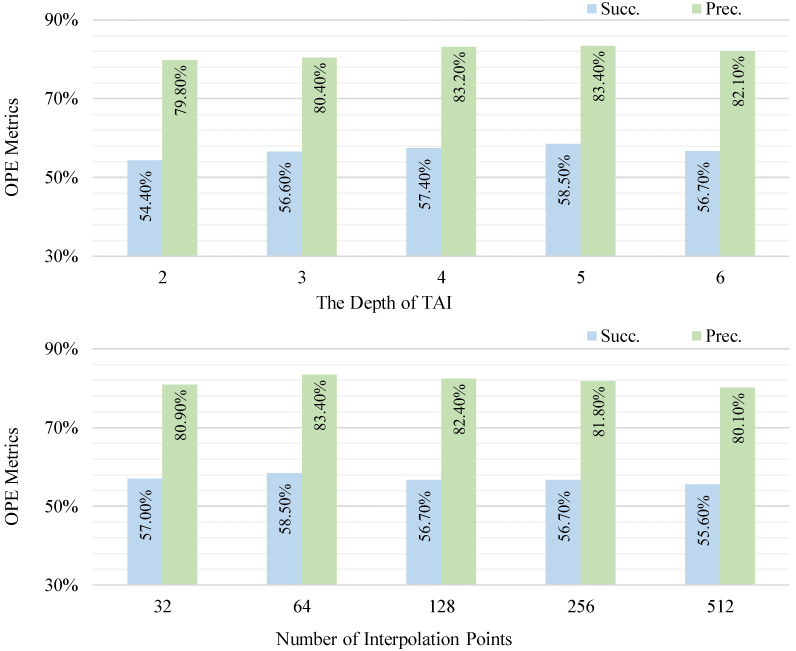
Exploration of the TAPM module. Two slides show the effect of the depth of self-attention and the number of interpolation points on the model, respectively.

TAPM module. In this part, we examine the impact of hyperparameters in the TAPM module on the tracking results. As shown in Figure 8, the metric improves from 54.4/79.8 to 58.5/83.4 as the self-attention depth of the TAPM module increases from 0 to 5. However, beyond a depth of 5, a further increase will lead to a decline in the metric. Similarly, a higher number of interpolation points does not always mean better performance. Optimal results are observed when the number of interpolation points is approximately 64, beyond which the performance plateaus.

## 5. Limitations

The proposed model has two primary limitations. Firstly, the TAPM module’s capability to discern object shapes is not fully robust. While the interpolation points can approximate the general shape to a certain degree, they fall short in reconstructing details. This issue lies in twofold: the quality of the template is often compromised as it is derived from cropped data, which tend to be incomplete; and the reliance on Chamfer Distance constraints alone is insufficient for restoring details. Secondly, the adaptability of the model across datasets has been somewhat compromised in favor of enhanced target recognition. Specifically, our model, which is trained on 64-beam LiDAR datasets like KITTI, struggles to adjust to 32-beam LiDAR datasets like nuScenes. To address shape reconstruction limits, we plan to integrate geometric priors (e.g., pre-trained models on completion task) and adversarial training. For the cross-dataset, we will improve its adaptability by using synthetic LiDAR data or feature distillation.

## 6. Conclusions

In this work, we formalize the challenge of small object tracking in 3D LiDAR point clouds. Our analysis reveals a critical performance degradation in state-of-the-art trackers when applied to scaled-down small objects, attributable to sparse foreground feature representation and cumulative spatial information loss during erosion. To address these limitations, we propose two novel components: the target-awareness prototype mining (TAPM) module and the regional grid subdivision (RGS) module. The TAPM module enhances target discriminability by adaptively extracting complete geometric prototypes from sparse search regions and reconstructing target representations through iterative point interpolation. Concurrently, the RGS module mitigates convolutional erosion by refining low-resolution BEV maps into high-resolution grids via pixel-shuffle operations and Vision Transformer, thereby enabling precise localization of sub-voxel structures. Under scaled small-object settings, our method achieves mean Success rates of 60.4%, surpassing the benchmark by +5.4%. Crucially, our framework preserves baseline performance for standard-sized targets, demonstrating task-specific adaptability without compromising generalizability.

## Figures and Tables

**Table 1:** Comparison with mainstream methods under our scaling setting. We simulate small object tracking by scaling other categories of objects to the same size as pedestrians. Specifically, we scaled the car and van categories by a factor of 0.25 and the cyclist category by a factor of 0.5. In this way, the pixels occupied by all categories of objects in the bird’s eye view meet the definition of small objects. “ORIG” refers to the experiment results for original object size. “SC” is for objects scaled to small size. “GAP” refers to the score gap before and after the change in the experimental setting. The up/down arrows indicate an improvement or degradation in tracking performance.

Methods	Pedestrian	Car		Van		Cyclist		Mean		
	ORIG	ORIG	SC	ORIG	SC	ORIG	SC	ORIG	SC	GAP
Success Metric										
P2B	28.7	56.2	15.4	40.8	12.2	32.1	34.9	42.4	21.3	−21.1
BAT	42.1	60.5	16.2	52.4	10.2	33.7	17.9	51.2	26.9	−24.3
M2Track	61.5	65.5	22.4	53.8	8.6	73.2	69.7	62.9	38.9	−20.5
STNet	49.9	72.1	60.5	58.0	48.1	73.5	69.4	61.3	55.0	−6.3
Ours	58.5	71.5	63.5	60.3	51.1	73.0	73.8	64.9	60.4	−4.5
Improvement	↑8.6	↓0.6	↑3.0	↑2.3	↑3.0	↓0.5	↑4.3	↑3.6	↑5.4	↑1.8
Precision Metric										
P2B	49.6	72.8	13.3	48.4	4.7	44.7	53.5	60.0	29.1	−30.9
BAT	70.1	77.7	20.9	67.0	9.0	45.4	29.7	72.8	41.3	−31.5
M2Track	88.2	80.8	28.0	70.7	5.9	93.5	87.8	83.4	53.4	−30.7
STNet	77.2	84.0	82.2	70.6	77.8	93.7	96.5	80.1	79.9	−0.2
Ours	83.4	84.0	84.6	74.9	83.4	93.9	97.1	83.0	84.2	1.2
Improvement	↑6.2	−0.0	↑2.4	↑4.3	↑5.6	↑0.2	↑0.6	↑2.9	↑4.3	↑1.4

**Table 2:** Comparison with the state of the arts on the KITTI datasets. The results in the cells represent the “Success/Precision” of the corresponding method (row) under the corresponding category (column). The red means the highest Success, and the mint indicates the second.

Methods	Car (6424)	Pedestrian (6088)	Van (1248)	Cyclist (308)	Mean
Success Metric (%)					
SC3D	41.3	18.2	40.4	41.5	31.2
P2B	56.2	28.7	40.8	32.1	42.4
3DSiamRPN	58.2	35.2	45.7	36.2	46.7
LTTR	65.0	33.2	35.8	66.2	48.7
MLVSNet	56.0	34.1	52.0	34.3	45.7
BAT	60.5	42.1	52.4	33.7	51.2
PTT	67.8	44.9	43.6	37.2	55.1
V2B	70.5	48.3	50.1	40.8	58.4
PTTR	65.2	50.9	52.5	65.1	57.9
STNet	72.1	49.9	58.0	73.5	61.3
M2Track	65.5	61.5	53.8	73.2	62.9
Trans3DT	73.3	53.5	59.2	46.3	62.9
Ours	71.5	58.5	60.3	73.0	64.9
Precision Metric (%)					
SC3D	57.9	37.8	47.0	70.4	48.5
P2B	72.8	49.6	48.4	44.7	60.0
3DSiamRPN	76.2	56.2	52.9	49.0	64.9
LTTR	77.1	56.8	45.6	89.9	65.8
MLVSNet	74.0	61.1	61.4	44.5	66.7
BAT	77.7	70.1	67.0	45.4	72.8
PTT	81.8	72.0	52.5	47.3	74.2
V2B	81.3	73.5	58.0	49.7	75.2
PTTR	77.4	81.6	61.8	90.5	78.1
STNet	84.0	77.2	70.6	93.7	80.1
M2Track	80.8	88.2	70.7	93.5	83.4
Trans3DT	84.7	79.8	70.5	56.5	80.7
Ours	84.0	83.4	74.9	93.9	83.0

**Table 3:** Comparison with state of the arts on nuScense datasets. The results in the cells represent the “Success/Precision” of the corresponding method (row) under the corresponding category (column). * means the results of migrating the model to the same environment as ours.

Methods	Car (15,578)	Pedestrian (8019)	Truck (3710)	Bicycle (501)	Mean
Success Metric (%)					
SC3D	25.0	14.2	25.7	17.0	21.8
P2B	27.0	15.9	21.5	20.0	22.9
BAT	22.5	17.3	19.3	17.0	20.5
V2B	31.	17.3	21.7	22.2	25.8
STNet	32.2	19.1	22.3	21.2	26.9
Trans3DT	31.8	17.4	22.7	18.5	26.2
P2B *	24.1	16.5	18.8	17.5	21.1
M2Track *	27.2	16.4	20.1	16.9	23.0
STNet *	25.5	14.9	18.9	17.0	21.4
Ours	25.6	15.3	12.7	17.5	20.8
Precision Metric (%)					
SC3D	27.1	17.2	21.9	18.2	23.1
P2B	29.2	22.0	16.2	26.4	25.3
BAT	24.1	24.5	15.8	18.8	23.0
V2B	35.1	23.4	16.7	19.1	29.0
STNet	36.1	27.2	16.8	29.2	30.8
Trans3DT	35.4	23.3	17.1	23.9	29.3
P2B *	24.6	20.0	13.1	18.9	21.6
M2Track *	28.3	18.9	16.5	16.6	23.8
STNet *	27.0	16.3	13.3	16.4	21.9
Ours	27.5	17.4	18.5	18.4	23.2

**Table 4:** Ablation studies of different voxel sizes. “STNet-0.2” indicates that the voxel size was set to 0.2 m for the experiment with other the settings unchanged.

Methods	Car (6424)	Pedestrian (6088)	Van (1248)	Cyclist (308)	Mean
Success Metric					
STNet-0.2	70.8	55.4	39.0	71.6	61.3
STNet-0.3	70.5	51.4	56.5	72.9	61.0
STNet-0.4	69.2	46.0	58.0	73.3	58.3
Ours-0.2	71.5	58.5	60.3	73.0	64.9
Ours-0.3	71.4	54.5	60.0	73.2	63.1
Ours-0.4	69.0	50.7	59.7	74.0	60.3
Precision Metric					
STNet-0.2	82.6	79.9	45.7	93.9	78.4
STNet-0.3	82.7	78.8	67.0	94.0	79.9
STNet-0.4	82.0	74.0	68.1	94.2	77.6
Ours-0.2	84.0	83.4	73.9	93.9	83.0
Ours-0.3	84.2	81.1	73.2	94.1	82.1
Ours-0.4	81.7	78.1	72.7	94.5	79.6

**Table 5:** The effectiveness of the proposed module. ✓ and \ refer to the w/o corresponding module, respectively. The values show the results tested on the pedestrian category.

TAPM Module	PixelShuffle	ViT Layer	Success	Precision
\	\	\	51.6	73.6
✓	\	\	51.7	74.9
\	✓	\	49.8	70.7
\	\	✓	56.0	80.7
✓	✓	\	54.2	75.9
\	✓	✓	56.4	82.4
✓	\	✓	56.4	81.5
✓	✓	✓	58.5	83.4

## Data Availability

The KITTI tracking dataset is available at http://www.cvlibs.net/download.php?file=data_tracking_velodyne.zip (accessed on 2 June 2025), and the nuScenes dataset is available at https://www.nuscenes.org/download (accessed on 2 June 2025). For the reported results, one can obtain it by request to corresponding author.
